# Prediction of residual disease using circulating DNA detection after potentiated radiotherapy for locally advanced head and neck cancer (NeckTAR): a study protocol for a prospective, multicentre trial

**DOI:** 10.1186/s12885-023-11136-2

**Published:** 2023-07-04

**Authors:** Angeline Ginzac, Marie-Céleste Ferreira, Anne Cayre, Clément Bouvet, Julian Biau, Ioana Molnar, Nicolas Saroul, Nathalie Pham-Dang, Xavier Durando, Maureen Bernadach

**Affiliations:** 1 Division de Recherche Clinique, Clermont Auvergne University, INSERM, U1240, Molecular Imaging and Theranostic Strategies, Jean PERRIN Center, 58, Rue Montalembert, 63011 Clermont-Ferrand, France; 2 Clinical Investigation Center, UMR501, 63011 Clermont-Ferrand, France; 3 Clinical Research Division, Delegation for Clinical Research and Innovation, Jean PERRIN Center, 63011 Clermont-Ferrand, France; 4 OncoGènAuvergne Laboratory, Pathology Unit, Jean PERRIN Center, Clermont-Ferrand, 63011 France; 5 Nuclear Medicine Department, Jean Perrin Center, Clermont Ferrand, 63011 France; 6 Radiotherapy department, Jean PERRIN Center, 63011 Clermont-Ferrand, France; 7 Department of Otorhinolaryngology - Head and Neck Surgery, Gabriel Montpied University Hospital Center, 63000 Clermont-Ferrand, France; 8 Department of Maxillofacial and Plastic Surgery, Estaing University Hospital Center Clermont-Ferrand, 63000 Clermont-Ferrand, France; 9 Medical Oncology Department, Jean PERRIN Center, 63011 Clermont-Ferrand, France

**Keywords:** Head and neck cancer, Potentiated radiotherapy, Residual disease, Circulating DNA, HPV-HR

## Abstract

**Background:**

Sensitive and reproducible detection of residual disease after treatment is a major challenge for patients with locally advanced head and neck cancer. Indeed, the current imaging techniques are not always reliable enough to determine the presence of residual disease. The aim of the NeckTAR trial is to assess the ability of circulating DNA (cDNA), both tumoral and viral, at three months post-treatment, to predict residual disease, at the time of the neck dissection, among patients with partial cervical lymph node response on PET-CT, after potentiated radiotherapy.

**Methods:**

This will be an interventional, multicentre, single-arm, open-label, prospective study. A blood sample will be screened for cDNA before potentiated radiotherapy and after 3 months if adenomegaly persists on the CT scan 3 months after the end of treatment. Patients will be enrolled in 4 sites in France. Evaluable patients, i.e. those with presence of cDNA at inclusion, an indication for neck dissection, and a blood sample at M3, will be followed for 30 months. Thirty-two evaluable patients are expected to be recruited in the study.

**Discussion:**

The decision to perform neck dissection in case of persistent cervical adenopathy after radio-chemotherapy for locally advanced head and neck cancer is not always straightforward. Although studies have shown that circulating tumour DNA is detectable in a large proportion of patients with head and neck cancer, enabling the monitoring of response, the current data is insufficient to allow routine use of this marker. Our study could lead to better identification of patients who do not have residual lymph node disease in order to avoid neck dissection and preserve their quality-of-life while maintaining their prospects of survival.

**Trial registration:**

Clinicaltrials.gov: NCT05710679, registered on 02/02/2023, https://clinicaltrials.gov/ct2/show/. Identifier with the French National Agency for the Safety of Medicines and Health Products (ANSM): N°ID RCB 2022-A01668-35, registered on July 15^th^, 2022.

## Background

With about 600 000 new cases per year, head and neck cancers (HNC) rank in 6^th^ position among the most common malignancies worldwide [1, 2]. More than half of the patients present a locally advanced stage at diagnosis [3]. The main risk factors for developing HNC are tobacco and alcohol consumption. High-risk papillomavirus (HPV) infection is also a cause of HNC, particularly for oropharynx cancer [4–6]. Despite treatment, 30 to 50% of patients relapse within 2 years and median survival does not then exceed one year [7–9]. The standard treatments for HNC are radiotherapy, surgery and chemotherapy [6].

Post-treatment follow-up of patients requires, among other things, a thorough clinical examination and repeated imagery investigations. The main difficulty lies in the interpretation of the results of these examinations as a result of post-operative and post-radiation changes that are difficult to distinguish from residual or recurrent tumour [10, 11].

The persistence of residual disease at the end of radiotherapy treatment is a major prognostic issue. 18F-fluorodeoxyglucose (FDG) positron emission tomography-computed tomography (PET-CT)-guided surveillance, with a negative predictive value of 95–97%, has proven to be non-inferior to cervical curage in CETEC with residual adenomegaly after radio-chemotherapy [12, 13]. Cervical curage is now indicated only if the response assessed by PET-CT is only partial. Nevertheless, the ability of PET-CT to predict residual disease is unsatisfactory because of a high frequency of false positives, due to inflammatory remodelling, with a positive predictive value of about 20–50% [14].

Currently, up to 50% of patients have residual adenomegaly after treatment, and only 30% have viable disease at the time of neck dissection [15, 16]. However, the acute and late complications to which patients who have undergone adenectomy are exposed are not negligible and can damage their quality-of-life (haemorrhage, scar disunion, alteration of swallowing function, etc.) [17–19].

Sensitive and reproducible detection of residual disease after treatment is a major issue for these patients. This is the context of our study, which aims to evaluate the prediction of residual disease using circulating DNA detection after potentiated radiotherapy for locally advanced head and neck cancer.

## Methods/design

### Study design

This interventional study is designed as a multicentre, single-arm, open-label, prospective study. The study has been registered on Clinicaltrials.gov (NCT05710679, 02/02/2023).

We expect to enrol 32 evaluable patients, i.e. with an indication for neck dissection and a blood sample at M3, to meet the primary objective. The enrolment period is expected to be 36 months. Each patient will be followed until two years after radiotherapy, reaching a total of 30 months.

### Coordination and participating institutions

The Centre Jean PERRIN (Clermont-Ferrand, France) sponsors the NeckTAR trial and is responsible for coordination, trial management, data management and trial monitoring.

This multicentre study is currently being conducted in 4 sites in France. The list of the study sites is available on https://clinicaltrials.gov/ct2/show/NCT05710679.

### Study objectives and endpoints

#### Primary objectives and endpoint

The primary objective of the study is to assess, at three months post-treatment, the ability of cDNA, tumoral and viral, to predict residual disease at the time of neck dissection among patients with partial cervical lymph node response after potentiated radiotherapy.

**Fig. 1:**
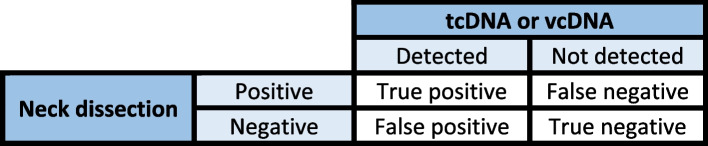
Confusion diagram

All patients with an indication for neck dissection will provide a blood sample for cDNA testing. To conclude on the diagnostic performance of post-treatment cDNA detection, we will compare the presence or absence of cDNA in the blood sample and the presence or absence of residual disease at the time of neck dissection (Fig. 1).

#### Secondary objectives and endpoints

Assessment of the cDNA detection rate among patients with residual adenomegaly after treatment. This will be evaluated according to cDNA detection and response on CT-scan.

Assessment of the relationship between cDNA detection and metabolic lymph node response on PET scan after treatment among patients with residual adenomegaly. This endpoint will be evaluated according to cDNA detection and objective metabolic response at three months after potentiated radiotherapy.

Assessment of the prognostic value of cDNA detection 3 months after the end of potentiated radiotherapy for patients with residual adenomegaly. This endpoint will be evaluated using overall survival and progression-free survival.

Assessment of the prognosis value of the presence of residual adenomegaly. This will be evaluated using overall survival and progression-free survival.

Assessment of the concordance of mutational profiles and HPV-HR genotypes between the primary tumour and cDNA at diagnosis. This endpoint will be evaluated on the basis of the mutational profiles from FFPE blocks and the inclusion blood sample.

Assessment of concordance between p16 immunohistochemistry and HPV-HR genotyping on the primary tumour.

Test of the concordance between real-time PCR and NGS on FFPE blocks for simultaneous detection and genotyping of HPV-HR at diagnosis.

Assessment of the relationship between ctDNA and cvDNA detection at diagnosis and the clinical, paraclinical and pathological features of the cancer.

Assessment of inter-observer reproducibility of the interpretation of SUVmax measures of residual cervical adenomegaly. A centralized review of the initial and three months post-treatment PET-CT will be performed by the sponsor’s nuclear medicine department.

### Participant eligibility

The exhaustive list of selection criteria is presented in Table 1. Patients with a recent diagnosis of locally-advanced head and neck cancer with lymph node involvement and never previously treated and for whom potentiated radiotherapy is indicated are eligible. After the signature of informed consent, a blood sample will be taken (2 tubes of 9 mL) and a FPPE block (from the tumour or a lymph node biopsy) will be recovered and sent to the sponsor. Centralized analyses will be conducted to identify specific tumour mutations on FFPE blocks and to search for the presence of cDNA in the blood sample. Patients for whom neither specific tumour mutations nor cDNA is found will be considered as screening failures.

**Table 1:** Selection criteria

Inclusion criteria	Non-inclusion criteria
Age ≥ 18 and ≤ 80 years old	Tumor of the nasopharynx, sinuses, nasal cavity, salivary glands or thyroid cancer
Epidermoïd carcinoma histologically confirmed, never treated, with lymph node envolvment	Treatment with radiotherapy only
Stage III (N1), stage IVa (minimum N1) or IVb, resectable but not operated or unresectable, with potentiated radiotherapy indication	Contraindication for neck dissection
Oral cavity, oropharynx, hypopharynx or larynx, cervical adenopathies without a primitive	Metastatic disease (stage IVc)
Tumoral sample FFPE available before the treatment initiation	History of treatment for a head and neck cancer
Presence of cDNA in the initial blood sample	History of other cancer within 3 years (except carcinoma in situ, basal cell skin carcinoma, localized prostate cancer Gleason 6)
Tumor-specific variant detected on FFPE and leucocytes	Pregnant or breastfeeding woman
Written informed consent signed	Patient under guardianship or curatorship
Affiliation to the French social security system	Psychological disorder (cognitive disorders, mental alertness, etc*.*) or social (deprivation of liberty by judicial or administrative decision) or geographical reasons that may compromise medical monitoring of the trial or compliance with treatment

### Blood sample for cDNA research

Each participant will provide a blood sample at inclusion, before the initiation of potentiated radiotherapy. Then, at the three-month evaluation of the disease, a second blood sample will be collected for the patients with residual lymph nodes on the scan.

After DNA extraction, a specific panel, defined previously and including the main mutation of head and neck cancer and HPV, will be applied to each of the samples to search for specific HNC mutations on ctDNA.

### Study procedures and participant timeline

**Fig. 2:**
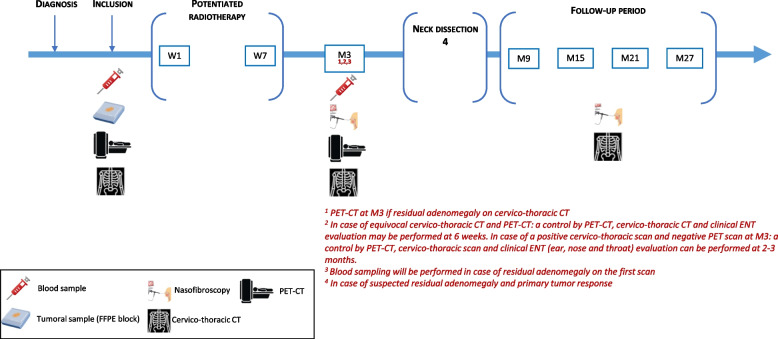
Scheme of the study

A SPIRIT flow diagram gives an overview of the study procedures (Table 2). After the inclusion visit, patients will be monitored twice during potentiated radiotherapy: at the beginning and at the end of radiotherapy. A visit is then planned three months after the end of radiotherapy. The other follow-up visits will be carried out every 6 months (M9, M15, M21, M27). The diagrammatic illustration of the study is presented in Fig. 2.

**Table 2:** Spirit flow diagram

		**Potentiated radiotherapy**		**Follow-up visits**				
**Assessment**	Baseline	W1	W7	M3	M9	M15	M21	M27
**Day**	-30 à 0	0 à 7	42 à 49					
*Medical history*	x							
*Clinical examination*	x	x	x	x	x	x	x	x
*Blood sample for cDNA research*	x			x^a^				
*Cervico-thoracic scan*	x			x	x	x	x	x
*PET-CT scan*	x			x^a^				
*Histological examination of the tumor or a lymph node*	x							
*Mutation profiling by NGS on FFPE, plasma and leukocytes*	x							
*p16 immunohistochemistry on FFPE*	x							
*HR-HPV genotyping by real-time PCR on FFPE*	x							
*HR-HPV genotyping by NGS on FFPE*	x							
*Doses received, fractionation for radiotherapy*			x					
*Data on the potentiating treatment received*			x					

*W* Week, *M* Month, *FFPE* Formol fixed paraffine embedded, *PET-CT* positon-emission tomography computed-tomography, cDNA circulating DNA, PCR Polymerase chain reaction, *HR-HPV* High risk human papilloma virus

^a^*In case of residual cervical adenomegaly at cervico-thoracic scan*

### Statistical analysis

#### Sample size calculations

Since the negative predictive value (NPV) of PET-CT to detect residual nodal disease in the context of our study is known to be very good (95–97%), we choose to focus on the group of patients with an indication for salvage adenectomy. The detection of ct/cvDNA should enable the identification of patients for whom this adenectomy is indeed necessary. This question means assessing the positive predictive value (PPV) of the ct/cvDNA predictor (detectable versus non-detectable) in relation to the lymph node dissection result (positive versus negative). For sample size determination, we hypothesise that the PPV of ct/cvDNA is at least 85% (the value obtained by Tanaka et al*.* being 100% from a calculation performed on 6 patients with detectable cvDNA [20]). Given an estimated 50% positive dissection rate among patients with an indication for salvage adenectomy, the inclusion of 28 patients is required to ensure a power of 80% at an alpha risk of 5% to show a PPV is greater than 50% (value taken as reference, equal to the upper limit of the imagery PPV estimate). This sample size will also enable the estimation of the NPV with a 95% confidence interval (CI) (Wilson) with a half-length equal to 16.5% (for an expected NPV of 89.7% obtained by Tanaka et al*.* [20]). To ensure the inclusion of 28 patients with adenectomy indication, n = 56 patients are required, since the proportion of patients with this indication after imagery following potentiated radiotherapy is estimated to be about 50% in our study population. Finally, assuming a low attrition rate of 10% (the time between inclusion and end of treatment being relatively short), n = 63 patients are required. Taking into account the additional risk that only 85% of the patients will have detectable ct/cvDNA [21], we estimate that it will be necessary to screen around 75 patients.

Recruitment will be complete once 32 patients with an indication for neck dissection are included and evaluable (this sample size takes into account the 10% attrition rate).

### Data analysis

#### Analyses of the primary endpoint

The primary analysis of the trial is the estimation of the PPV of ctDNA/cvDNA detection at 3 months post-treatment in predicting the outcome of neck dissection. The contingency table for this analysis is presented in Fig. 1. This analysis will be performed on the population undergoing neck dissection. A point estimate and CI will be provided and the PPV will be compared to the reference value of 50% using an exact binomial test.

#### Analyses of secondary endpoints

Secondary analyses will be performed primarily on all patients (regardless of indication for neck dissection), and also in the subgroup of patients with an indication for neck dissection (as for the primary objective).

The relationship between ctDNA/cvDNA detection at 3 months post-treatment and the treatment failure rate, as well as the relapse rate during follow-up, will be evaluated using the classic diagnostic performance measures calculated on contingency tables (including PPV, NPV, sensitivity, specificity, overall accuracy). In addition, for concordance analyses (of binary variables) between ctDNA/cvDNA and primary tumour, between p16 IHS and real-time PCR, and between real-time PCR and NGS, Cohen's kappa method will also be used.

Survival will be analysed using Kaplan–Meier curves (and log-rank test if relevant). The prognostic value of the presence of residual adenomegaly will be assessed using a Cox model. Other prognostic factors for survival will be investigated using firstly univariate Cox models, and then multivariate models to assess the prognostic value of the presence of residual adenomegaly in particular, and to identify possible confounding factors. For patients with ct/cvDNA collection and analysis at 3 months after the end of treatment, the prognostic impact of ct/cvDNA detection at 3 months in particular will be analysed.

The relationship between ctDNA/cvDNA detection at diagnosis and the clinical, para-clinical and pathological characteristics of the cancer will be performed using McNemar's test, Wilcoxon's signed rank test, Spearman's correlation and multiple linear or logistic regression models, depending on the type of data.

Inter-observer reliability will be assessed using Cohen's kappa method on three-level criteria (positive, doubtful lymph node involvement, or negative).

### Data management and monitoring

The data collected for the study will be registered on an electronic case report form (eCRF) (Ennov Clinical). Data will be pseudonymised using a specific identification code. Each centre will manage a table for the correspondence between specific identification codes and patient identity. The access to the eCRF is limited to the investigators, clinical research associates (CRA), project manager, biostatistician, data-manager and monitor CRA. The monitoring of the data will be performed by the monitor CRA mandated by the sponsor and according to the study data monitoring plan. On-site and off-site monitoring visits will be conducted and monitoring reports will be drafted to ensure traceability.

### Independent data monitoring committee (IDMC)

An independent trial monitoring committee will be set up. It will be composed of three members with specific areas of expertise (biostatistician, oncologist, head and neck surgeon). This committee will meet after the analysis of the results of the neck dissection for the first 20 eligible patients, i.e. who had a neck dissection and a blood sample at M3.

The main objective of this committee will be to ensure that the assumptions used to calculate the number of subjects are met. A readjustment of the number of eligible subjects needed to meet the objective can be performed subject to the opinion of this committee.

The recruitment rate will also be analysed in order to judge the feasibility of the study (recruitment rate compatible with its continuation).

## Discussion

The improvement in detection of residual disease after treatment for advanced head and neck cancer is a current challenge. Our study is part of this context and aims to more efficiently identify patients who do not have residual disease. This will avoid them having undergo neck dissection and will preserve their quality-of-life while maintaining their survival.

As underlined by Yang and colleagues, in the studies focused on ctDNA mutation in the plasma of HNSCC patients, there are very few analyses of the concordance with tumour samples, and few analyses of sensitivity or specificity [22]. One of the strengths of our study is that the proposed analytical technology is a massive parallel sequencing or NGS capture panel integrating the main genes involved in HNSCC as well as the E7 genomic region of HR-HPV. This panel will allow on the one hand simultanous identification of informative tumor variants and detection/genotyping of HR-HPV on FFPE tissue; on the other hand their respective follow-up on circulating DNA. Evaluation of specificity and sensitivity of techniques will also be performed before analyzing patient samples.

## Trial status

The study was approved by the ethics committee on December 29^th^, 2022 (CPP Ouest-1). The recruitment is expected to begin in the first half of 2023. Follow-up and data collection will be complete in 2028.

## Data Availability

Not applicable.

## Abbreviations

cDNA: Circulating deoxyribonucleic acid
CI: Confidence interval
ctDNA: Circulating tumour deoxyribonucleic acid
cvDNA: Circulating viral deoxyribonucleic acid
CRA: Clinical research associate
eCRF: Electronic case report form
FFPE: Formol-fixed paraffin-embedded
HNC: Head and neck cancer
HR-HPV: High risk human papilloma virus
NGS: Next generation sequencing
NPV: Negative predictive value
PCR: Polymerase chain reaction
PET-CT: Positon emission tomography computed tomography
PPV: Positive predictive value
SUVmax: Maximum standardized uptake value
